# Is this to be another project that fizzles out? Using the i‐PARIHS framework to evaluate implementation of a mentoring programme

**DOI:** 10.1111/jan.16041

**Published:** 2024-01-04

**Authors:** Lena Nyholm, Lena Gunningberg, Eva Jangland

**Affiliations:** ^1^ Department of Surgical Sciences, Nursing Research Uppsala University, Uppsala University Hospital Uppsala Sweden; ^2^ Department of Public Health and Caring Sciences Uppsala University Uppsala Sweden

**Keywords:** case study, facilitators, implementation, leadership, mentoring programme

## Abstract

It is well‐known that the implementation of evidence into clinical practice is complex and challenging. The integrated Promoting Action on Research Implementation in Health Services (i‐PARIHS) framework conceptualizes successful implementation of evidence into practice. As the implementation of the mentoring programme proved to be a challenge, it seemed valuable to retrospectively study the implementation process using a framework like the i‐PARIHS.

**Aim:**

The aim of this study was to evaluate implementation of a multifaceted mentoring programme for bedside nurses using the i‐PARIHS framework, to identify factors that influenced the implementation.

**Design:**

A secondary analysis of qualitative data using the i‐PARIHS framework as the theoretical lens.

**Method:**

A directed content analysis was performed, driven theoretically by the i‐PARIHS framework. The analysis focused separately on (a) characteristics of the innovation and (b) successful and hindering factors in the implementation process.

**Results:**

The results showed that successful factors influencing implementation of the mentoring programme included supportive and actively involved formal leaders and supervisors at the unit level. A major hindering factor was lack of resources in the form of personnel, time and money. A lack of facilitators, particularly experienced facilitators, throughout the organization hindered implementation. The i‐PARIHS framework offered a structured how‐to guide to identify factors that influenced the implementation process.

**Conclusion:**

Implementation of the mentoring programme was a challenge for the organization. Investment into implementation should continue, with a more structured facilitation process. A structured and prioritized management system, including supportive leadership at the unit level, should be established by the hospital board.

**Implications for the profession:**

There is a need for experienced facilitators throughout the organization. This is crucial to achieve sustainability in the mentoring programme and ensure that the large investments of staff resources and money do not fizzle out.

**Impact:**

**What problem did the study address?**

Implementing a mentoring programme for nurses in a large university hospital proved to be a challenge. Therefore, it seemed valuable to retrospectively study the implementation process using a framework like the i‐PARIHS.

**What were the main findings?**

A lack of facilitators, particularly experienced facilitators, throughout the organization hindered the implementation. The i‐PARIHS framework offered a structured how‐to guide to identify factors that influenced the implementation process.

**Where and on whom will the research have an impact?**

Our findings are important for leaders on all levels in a hospital setting, including the hospital board, heads of departments and nurse managers.

**Reporting Method:**

Consolidated criteria for reporting qualitative research (COREQ): a 32‐item checklist for interviews and focus groups is used.

**Patient or Public Contribution:**

No patient or public contribution.

## 1 INTRODUCTION

It is well‐known that the implementation of evidence into clinical practice is complex and challenging (Kitson & Harvey, 2016). Two decades ago, the Promoting Action on Research Implementation in Health Services (PARIHS) framework was developed by Kitson et al. (1998). It conceptualizes successful implementation of evidence into practice. A recent systematic review, including 367 articles, describes PARIHS as a well‐established framework and shows how it has been operationalized in the scientific literature (Bergström et al., 2020). It reported that 32% of the included articles used the PARIHS in planning and delivering an intervention, 50% in data analysis, 55% in evaluation of study findings and/or 37% in some other way. Further analysis showed that its application was often partial and generally not well‐elaborated. Findings also pointed at difficulties in using the framework, such as lack of guidance on key steps to overcome barriers and support implementation (Bergström et al., 2020).

The framework was refined in 2015 and the new framework, the integrated PARIHS or ‘i‐PARIHS’ framework, contains a set of practical instructions for people wishing to use it (Harvey & Kitson, 2015). Facilitation, innovation, recipients and context are the core constructs of the i‐PARIHS framework (Kitson & Harvey, 2016). *Facilitation* is the active element when assessing, aligning and integrating the other three constructs. Three core facilitation roles have been identified (novice, experienced and expert). *Innovation* describes the focus or content of the implementation. *Recipients* include staff, support services and patients who will be directly involved in and affected by the implementation process. *Context* refers to local and organizational factors, and their potential impact on the success of the implementation process. Supportive and engaged leadership is pivotal in the implementation of new evidence into clinical practice (Bianchi et al., 2018). Factors that influence leaders to be proactive and successful are graduate‐level education, years of leadership experience, and ability to create an empowering environment (Bianchi et al., 2018).

## 2 BACKGROUND

The World Health Organization has reported a worldwide shortage of registered nurses. Before the COVID‐19 pandemic, the shortage was estimated to be 6 million registered nurses. It is estimated that 13 million registered nurses need to be recruited within the coming decade. Healthcare organizations need long‐term competence planning strategies to retain registered nurses, prevent their premature departure from the profession and use their competencies so that people can get the care they need (Stewart, 2022; World Health Organization, 2020).

To address this problem, a mentoring programme was implemented in a large Swedish university hospital. There are few published conceptual definitions of mentor and supervisor. Mills et al. found in a literature review that these two concepts are close to each other and describes that both concepts implies long‐term relationships with the persons being supervised and where progression is sought (Mills et al., 2005). The supervision can be one‐on‐one or within a group. In most cases, even the supervisor gets personal development and increased knowledge (Mills et al., 2005). The programme in the present study focused on supporting newly graduated nurses' transition to practice and senior nurses' professional development as supervisors. It appeared to be a way to smooth the transition for newly graduated nurses and to ensure senior nurses were challenged to grow in their role (Jangland et al., 2021). The programme was based on the latest evidence on how to retain bedside nurses in the profession and also how to avoid burnout and turnover during the early part of the career (Ahlstedt et al., 2019; Brook et al., 2019; Hayes et al., 2012; Rudman et al., 2020). It was also guided with a theoretical nursing model as evidence (Dock & Stewart, 1920; Eriksson et al., 1999). Despite this, it was a challenge to implement the programme to a larger extent across the hospital.

### 2.1 Rationale

A strategic, step‐by‐step implementation plan, involving nurse managers and bedside nurses, was used to implement a mentoring programme, but no implementation framework was actively used. As implementing the mentoring programme proved to be a challenge, it seemed valuable to retrospectively study the implementation process using a framework like the i‐PARIHS.

## 3 THE STUDY

### 3.1 Aim

To evaluate implementation of a multifaceted mentoring programme for bedside nurses using the i‐PARIHS framework, and to identify factors that influenced the implementation.

## 4 METHODS

### 4.1 Design

A secondary analysis of qualitative data (Beck, 2016) using the i‐PARIHS framework as the theoretical lens (Harvey & Kitson, 2015). When the interviews were analysed for the previous article, no attention was paid to the implementation process. Therefore, the interviews were re‐analysed, with a focus on that process.

### 4.2 Context

The study was conducted at a large Swedish university hospital. The hospital has 46 somatic units, approximately 800 patient beds and 8600 employees, including 2300 registered nurses.

### 4.3 The mentoring programme

The aim of the mentoring programme was to give senior nurses opportunity for professional development at the same time as they passed on their experiences to newly graduated nurses (Eriksson et al., 1999; Jangland et al., 2021). Three types of supervisors were implemented based on the head–heart–hand model:

*Clinical supervisors* provided practical and situational guidance in clinical nursing work. This included ensuring that the department's routines were followed, guidance in new work routines and prioritizing tasks.

*Group supervisors* provided group discussions where 5–10 newly graduated nurses shared experiences and reflected together on different clinical situations.

*Theoretical supervisors* provided support in theoretical development by introducing nursing rounds in clinical practice where patients' care needs were systematically assessed, discussed and evidence‐based nursing interventions prescribed.

The mentoring programme is described in a previously published article by Jangland et al. (2021).

### 4.4 Sample and setting

Five units were strategically selected based on their implementation of one or several parts of the mentoring programme. The 5 units included represented five different specialties in somatic care of adults and children. Interviews were conducted with the nurse manager and supervisors at each unit (for details, see Table 1). All invited nurse managers (*n* = 5, interviewed twice), supervisors (clinical supervisors [*n* = 8]), group supervisors (*n* = 4) and theoretical supervisors (*n* = 3) agreed to participate. In total, 24 interviews were conducted (one clinical supervisor was also a group supervisor). Conceivable participants were first contacted by e‐mail and included until data saturation was achieved. A few invited declined to participate. No reason for non‐participation was asked for.

**TABLE 1:** Outline of the characteristics of the included units.

	Unit A	Unit B	Unit C	Unit D	Unit E
Type of patients	Neonatal	Adult	Adult	Paediatric	Adult
Type of care	Intensive care unit Neonatal	Intensive care Cardiac	Step‐down unit Neurosurgery	General ward Orthopaedic, urologic and neurologic surgery	General ward Thoracic surgery
Employed registered nurses^a^ (*n*)	38	17	19	33	29
Supervisors' level of education	Registered nurses and clinical nurse specialists	Registered nurses	Registered nurses	Registered nurses and clinical nurse specialists	Registered nurses
Supervisors in 2019 when the interviews were performed	1 × CS^b^, ^d^	2 × CS^b^ 1 × GS^b^ 1 × TS^b^	1 × CS^b^ 1 × GS^b^ 1 × TS^b^	4 × CS^b^	1 × CS^b^ 1 × GS^b^ (from another unit)
Included	2 × CS^b^ 1 × GS^b^ 1 TS^b^	1 × CS^b^ 1 × GS^b^, ^c^ 1 × TS^b^	1 × CS^b^ 1 × GS^b^ 1 × TS^b^	2 × CS^b^	2 × CS^b^ 1 × GS^b^

^a^ Mean value of the number of registered nurses, converted to full time in the years 2017, 2018 and 2019.

^b^ CS, clinical supervisor; GS, group supervisor; TS, theoretical supervisor.

^c^ One clinical supervisor was also a group supervisor.

^d^ Fresh start with a newly educated clinical supervisor.

### 4.5 Data collection and procedure

Data on the characteristics of the innovation were collected retrospectively from hospital documents regarding the mentoring programme (i.e. project plan, plan for implementation and evaluation). The documents were retrieved from an archive at the hospital that is open to employees.

The first interview at each unit was conducted with the nurse manager. This interview included a visit to the unit to collect information about available competencies, recruitment of bedside nurses and other ongoing nursing‐related projects. In the next step, individual interviews were held with supervisors and newly graduated nurses (the latter were not included in this paper). Lastly, follow‐up interviews (focusing on implementation) were performed with the nurse managers. The interview questions were open‐ended, generated in accordance with the head–heart–hand model (Eriksson et al., 1999), and based on discussions within the research team. There were also specific questions to the nurse manager in the second interview about the implementation process such as ‘Could you please tell me about the main focus of the implementation at your unit?’. The last author (senior researcher with extensive experience of qualitative research) conducted all but two interviews; two interviews were conducted by a research assistant (a PhD student guided by the last author). All interviews were audio‐recorded, conducted in a conference room (supervisors) or office (nurse managers) at a time convenient for participants and lasted 27–70 min. Field notes were made after the interviews. Interview data were transcribed by professional secretaries and the research assistant verified the transcripts, but they were not returned to participants. Minor adjustments were made after the first two interviews, which are included in the study.

### 4.6 Data analysis

Hospital documents regarding the mentoring programme (i.e. project plan, plan for implementation and evaluation) were compiled to describe the innovation based on the i‐PARIHS, see Table 2. Author 1 individually coded the hospital documents using directed content analysis. The analysis was deductive and based on i‐PARIHS (characteristics of the innovation). All the authors then discussed the coding and how the results should be interpreted.

**TABLE 2:** Analysis process based on the i‐PARIHS (Harvey & Kitson, 2015).

C*haracteristics of the innovation* Author 1 individually coded the hospital documents regarding the mentoring programme (i.e. project plan, plan for implementation and evaluation).
b *Successful and hindering factors in the implementation process* Authors 1 and 3 individually read the interview transcripts word by word and highlighted all text that at first sight seemed related to the implementation process. Authors 1 and 3 individually coded the interviews in the pre‐determined categories based on the inner context: local level and recipients. The nurse managers' interviews were coded based on *The inner context: local level*. The supervisors' interviews were coded based on *Recipients*. The two separate codings of data were discussed and some minor revisions were made. Author 2 reads a selection of the interviews, and the coding of data was discussed. Some further minor adjustments were made. Throughout all coding, all authors met regularly to discuss the coding of data in relation to the i‐PARIHS.

**FIGURE 1:**
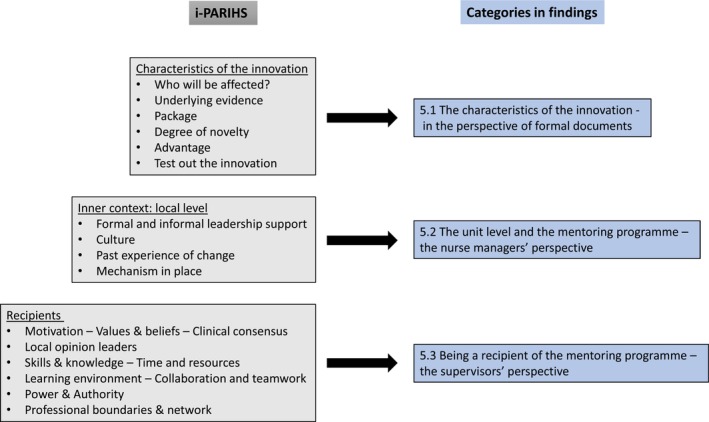
Outline of used parts of i‐PARIHS related to findings. *Source*: Kitson AL, Harvey G. Methods to succeed in effective knowledge translation in clinical practice. *Journal of Nursing Scholarship*. 2016;48(3):294–302.

The interviews were analysed by a directed content analysis (Hsieh & Shannon, 2005), driven theoretically by the i‐PARIHS framework (the inner context: local level and recipients), see Figure 1. The analysis focused on successful and hindering factors in the implementation process. Details of the analysis are presented in Table 2 and Figure 1. Authors 1 and 2 initially read all interviews. In the next step the interviews were read again and analysed in two rounds, first the interviews with nurse managers and then the interviews with the supervisors. Author 2 reads a selection of the interviews, and the coding of data was discussed. Some further minor adjustments were made.

Those who were interviewed were not given the opportunity to provide feedback on the results. The authors' preunderstandings, background, experiences and perspectives were discussed at research meetings.

All authors are female senior researchers with extensive experience in qualitative research and registered nurses with several years of clinical experience employed at the University. The first author credential is PhD, the second is professor of nursing and the last is associate professor of nursing. Two of the authors (LN and LG) were involved in the development and implementation of the mentoring programme. They had no previous relationships with the participants and no professional role in, or commitment to, the included units.

### 4.7 Ethical considerations

The study was performed in accordance with the Helsinki Declaration (World Medical Association, 2013). According to the Swedish Ethical Review Authority no ethical approval was required (No. 2019‐03102). All participants received information (verbal and written) were ensured confidentiality and provided written informed consent. The researchers were familiar with the hospital, but had no previous association with the participants. Nothing in the hospital documents could be linked to any individual person.

## 5 FINDINGS

The results are presented in three main categories in accordance with the i‐PARIHS. Aspects influencing the success of implementation of the mentoring programme for bedside nurses were identified and divided in successful and hindering factors. The category *Characteristics of the innovation* encompasses data from hospital documents regarding the mentoring programme. The category *The unit level and the mentoring programme* encompassed data from interviews with nurse managers and the *category Being a recipient of the mentoring programme* encompassed data from interviews with supervisors (Figure 1). Quotations reflecting the findings are integrated within the findings.

### 5.1 The characteristics of the innovation in the perspective of formal documents

The implementation of the multifaceted mentoring programme is described based on the i‐PARIHS framework's *Characteristics of the innovation*, see Table 3.

**TABLE 3:** Characteristics of the innovation in accordance with the i‐PARIHS (Harvey & Kitson, 2015).

Who will be affected?	Mainly newly graduated nurses, supervisors and nurse managers, but all other bedside nurses will eventually be affected, as well as patients
Underlying evidence	The mentoring programme was designed based on available scientific literature on the subject, in collaboration with newly graduated nurses, senior nurses and nurse managers as well as in consultation with *human resource* management and hospital management. The programme was based on the nursing theory ‘head–heart–hand’ model (Dock & Stewart, 1920; Eriksson et al., 1999)
Package	The project management distributed information about the project at meetings and in individual conversations with nurse managers. Written information was produced in the form of brochures and digital presentations. The project management also initiated and arranged meetings and invited expert speakers in the field to convince stakeholders at different levels in the hospital to prioritize the programme, help them understand its value and allocate time for implementation
Degree of novelty	The mentoring programme added a structure for knowledge transfer and an evidence‐based education for becoming a supervisor, based on nursing theory
Advantage	To improve the quality of the introduction of newly graduated nurses and simultaneously utilize the experience available among senior nurses. The intention was to secure the supply of qualified bedside nurses by retaining experienced nurses, reducing turnover and encouraging new recruitment
Test out the innovation	The mentoring programme was introduced throughout the hospital without a prior pilot study

### 5.2 The unit level and the mentoring programme—The nurse managers' perspective

#### 5.2.1 Formal and informal leadership support

The nurse managers reported a substantial shortage of bedside nurses, but prioritized introducing the mentoring programme because of the urgent need for supervision and support of newly graduated nurses. All nurse managers had extensive experience of leadership.

##### Successful factors

A successful factor in the implementation was nurse managers who had a well‐thoughtout plan on whom to recruit as supervisors, for example experienced, well‐educated bedside nurses who were motivated, with the capacity and competence to work independently. These nurse managers prioritized and had the foresight needed to succeed with scheduling. Despite this, it was a challenge to provide supervision to every newly graduated nurse needing it. One example from a thoughtout plan was a nurse manager who set aside time for the clinical supervisor to develop and adapt the mentoring programme to be implemented at the unit. Another example was a nurse manager who prioritized available resources so that an experienced nurse at the unit was recruited to take on the role as full‐time clinical supervisor. This decision by the manager was supported by the head of the department.

It became a real priority to free up Maria's time and free her from her duties in order for her to attend the mentoring programme and in any event we were already going to deal with it. Because if you're going to do something you might as well go all the way. There's no point in someone going through training and then not being able to use what they've learned. (Nurse manager 1)

Supporting the supervisors appeared to be one of the nurse managers' main keys to success. Some were always available for reflection and support when needed. Others had regular meetings following up on the progress of supervisors and how newly graduated nurses developed in their work. Recruiting new supervisors when needed was also described as a success factor for the project to sustainable over time. One nurse manager emphasized that being a supervisor is a difficult task. For example, it takes courage to give feedback.

##### Hindering factors

A major hindering factor was a lack of resources in the form of personnel, time and money. The mentoring programme was experienced as unclear and difficult to understand by some nurse managers. This is interpreted as that the nurse managers would have needed more support from the mentoring programme's project manager.

I think it is my responsibility to a very high degree. Absolutely. But the way everyday operations looks at the unit, it doesn't work. It's not that I don't want to, and it's not that the nurses themselves don't want to. But with the pace and the pressure, and since we always seem to just barely get things done, it doesn't work. (Nurse manager 2)

Another hindering factor was the lack of experienced nurses possible to recruit as an independent supervisor. In addition, most of the bedside nurses at the unit were newly graduated and unexperienced and in need of supervision.

So I really have very few people who can fill those shoes. Also, I think this is common in many units, that nurses don't stay on for very long. (Nurse manager 2)

In one unit, a job description was written about how the clinical supervisors should work, but in reality, it turned out to be something completely different. The clinical supervisors were told to supervise according to the programme, but in reality, they were also supervising students. Furthermore, as they were the most experienced, they often needed to take care of the most seriously ill patients at the same time. When a project, as in this case, is said to include supervision and time for reflection but proves not to, this leads to disappointment and frustration among both newly graduated nurses and supervisors.

We put a huge burden on them. So the ones we had from the start, they're gone now. (Nurse manager 2)

I think our clinical supervisors were given great training but as nurse manager and a unit, we didn't provide the right circumstances for them… (Nurse manager 2)

On the other hand, there were also supervisors who were given adequate time, but had not started the project and it was difficult to determine the reasons for this.

Some time must be allotted, but there's a big difference between giving a person the same amount of time to do something, and how much is actually accomplished. I mean it can be two completely different things depending on how much… how engaged you are in that particular mission. (Nurse manager 3)

#### 5.2.2 Culture

##### Successful factors

Many bedside nurses in the units were positive about the project, mainly because their workload was reduced when newly graduated nurses had a supervisor assigned to answer their many questions.

##### Hindering factors

A hindering factor was the problem of getting into the routine of having regular meetings for group supervision. This was mostly due to the newly graduated nurses who were invited feeling that they could not abandon responsibility for their patients. One nurse manager said that some of the new graduates were probably afraid that their colleagues would be upset if they left their duties for group supervision. Even if a nurse manager had encouraged them to take the time to attend such meetings, they did not participate.

We formed groups of eight nurses each. And I think the one that had the most members only had two. Only a few people could find the time to get away. And everyone really wanted to. They were all really looking forward to coming. But it's impossible to leave a unit that… you just can't leave your patients. There's no one to hand them over to. (Nurse manager 4)

Another obstructing factor was when a supervisor, who had full support from the nurse manager, was opposed by a unit's most experienced nurses. The reason for such actions was not clear, but one potential explanation mentioned was the opinion that the mentoring programme took time from patient care without improving anything. In the narratives, the nurse managers highlighted their responsibility to adapt the mentoring programme to their units, implementing it slowly and thoughtfully and investing in repeat information.

What happens to people who are stressed, which we see all the time, is that they become angry. They want to know what all their other colleagues are doing when they're away from their regular duties. It becomes hard to justify. (Nurse manager 2)

#### 5.2.3 Past experience of change

##### Successful factors

The nurse managers had past experience of implementing projects at their department. This experience included both successful and failed projects.

##### Hindering factors

No hindering factor could be identified in the category.

#### 5.2.4 Mechanism in place

##### Successful factors

A successful factor was to start the implementation process by evaluating the newly graduated nurses' requests, expectations and knowledge gaps. This was a way to adapt the supervision based on the needs of the newly graduated nurses. Another successful factor was that the nurse manager clearly confirmed who was to be supervised, which also gave the supervisor authority. This was exemplified in the narratives from one unit, where there was a planning schedule for staffing during day, evening and night shifts. A newly graduated nurse who was to be supervised by the clinical supervisor received a heart sticker next to their name, so it was clear to all that this person was to be supervised by the clinical supervisor during this work shift.

I put a heart sticker and then “Maria” next to the person's name on the planning chart so that they could see that ‘oh, right, Maria's coming with me that day.’ (Nurse manager 1)

##### Hindering factors

Not reaching out with information was a hindering factor in the implementation process. Information was presented at all staff meetings and was available in minutes from the meetings for those who did not attend. In addition, information about the mentoring programme was shared during the units' training days. Despite this, staff members stated that they had not received any information about the mentoring programme.

### 5.3 Being a recipient of the mentoring programme—The supervisors' perspective

#### 5.3.1 Motivation—Values and beliefs—Clinical consensus

One successful factor was that the supervisors perceived the mentoring programme as valuable and meaningful. The structure of the mentoring programme was consistent with their view of newly graduated nurses' needs for supervision. The experienced nurses found it stimulating and enjoyable to be supervisors. Some stated that they realized how important this was to the newly graduated nurses and that this motivated them. Moreover, they appreciated the opportunity to share their knowledge as well as learning new things. Occurrence of clinical consensus was not mentioned by anyone.

I think it's fun when I can see other people develop, watch them grow as individuals and in their profession – how they become more technically proficient, but also ground themselves, find security and observe how they become more comfortable. I'm both proud and happy when I see good interactions being performed in a professional manner. And then I think that maybe I had some small part to play in this, gently guiding them forward to their goal. (Clinical supervisor 1)

#### 5.3.2 Local opinion leaders

The local opinion leaders (nursing colleagues on the unit) were mostly found to be supportive. The bedside nurses at the units experienced a relief in their workload when newly graduated nurses had supervisors who supported them. This made most of the bedside nurses positive to the project. However, one supervisor felt that the colleagues opposed her.

So that they say that there's a huge difference during the shifts when I'm supervising because then all the questions are directed at me. So that they can work a bit more in peace. So it's… a bonus for them, too, I think. (Clinical supervisor 2)

#### 5.3.3 Skills and knowledge—Time and resources

A successful factor was that the supervisors saw themselves as having both the skills and the knowledge to manage the new task. All supervisors were satisfied with the education and had completed the training. Having several supervisors sharing the assignment was considered an advantage. This created an opportunity to talk, reflect and brainstorm with others familiar with the situation.

The amount of time and support provided was considered either a successful or a hindering factor. The clinical supervisors and the group supervisors felt that they were given enough time to complete their assignments, while the theoretical supervisors did not. The clinical supervisors stated they received support from the nurse managers, while the other supervisors experienced some support or no support from the nurse managers. The supervisors stated that they received some support from fellow supervisors.

#### 5.3.4 Learning environment—Collaboration and teamwork

The environment was mostly described as a successful factor regarding the implementation of the mentoring programme and other new projects, which could be interpreted as taking place in a learning organization. The majority of supervisors mentioned collaboration with their nurse managers. Clinical teachers and schedulers were also mentioned as important collaboration partners.

#### 5.3.5 Power and Authority

A successful factor that all supervisors mentioned was their being given power and authority from their nurse manager. Such authority was communicated to the staff with varying degrees of clarity. In some cases, it was very clear who was the supervisor and what they were supposed to do, while in other cases only a few of the staff members knew about the mentoring programme. One supervisor received a written assignment that described her duties.

Yes, but she [nurse manager] learned about our thought process and she really… She's also continued to push us, you know, ‘yes, but now let's do this’ and ‘you continue’ and ‘you're the ones responsible for this now and keeping track of it a bit’. (Clinical supervisor 1)

#### 5.3.6 Professional boundaries and network

It was a hindering factor that the supervisors did not perceive any boundaries and that they did not participate in any networks other than that organized by the mentoring programme's project management team. The supervisors felt, to varying degrees, that they received support from the mentoring programme's project management team. Some supervisors stated that they knew that there were follow‐up meetings and networking, but did not have the time to attend these meetings.

I've also been spitballing a bit with Lena and Sara [members of mentoring programme's project management] during this year. So the meetings we had here during the first year… in groups with them have been really, really good. And I've been e‐mailing them a bit on the side when problems have popped up. (Clinical supervisor 2)

## 6 DISCUSSION

Successful factors influencing the implementation of the mentoring programme was supportive and actively involved formal leaders and engaged supervisors at unit level, while the lack of facilitators, and in specific experienced facilitators, throughout the organization challenged the implementation. The i‐PARIHS framework offered a structured how‐to guide to identify factors that influenced the implementation process, see Figure 1.

Nurse managers who had well‐thoughtout plans on who could be recruited as supervisors, allocated time for implementation of the supervisor role and performed the implementation step by step, were found to succeed. Furthermore, these nurse managers were actively involved in the planning of the implementation and supported the supervisors in their new role. An engaged leadership style is confirmed as pivotal for successful implementation of evidence (Bianchi et al., 2018). Furthermore, in the i‐PARIHS framework, formal leadership (in this study: the nurse manager) is underlined as a crucial factor to create the prerequisites and culture for an innovation to be successfully implemented (Harvey & Kitson, 2015). However, it was hard for supportive and active nurse managers to succeed in scheduling all newly graduated nurses with a supervisor—as most bedside nurses at the units were new and in need of supervision. In addition, it was a challenge to recruit experienced nurses to take on the role as supervisor since this competence were not available to the extent needed. This highlights the complexity of implementation in health care that also led to the refinement of the PARIHS framework, where three core facilitation roles were identified (novice, experienced and expert) (Harvey & Kitson, 2015). Hence, the i‐PARIHS underlines the facilitator as the most crucial aspect for successfully implementing new innovations—like the mentoring programme—into everyday practice. In this case, the role as facilitator was given to the nurse manager, but this was not communicated clearly enough.

The nurse managers described a need of more information from the project management team on how to adapt the programme to their unit, given their limited resources in the form of time and competence. The implementation process at the hospital level included a long preparatory phase where nurse managers were informed and supported at several occasions and a structured education was offered to supervisors (Jangland et al., 2021). However, our evaluation reveals a need of more support to the nurse managers from the project management team: allocating experienced facilitators for starting up the project and adapting it to each unit, as well as educating novice facilitators. Based on the recommendations in the i‐PARIHS framework (Kitson & Harvey, 2016), the nurse managers were important stakeholders in this large implementation project—one of several projects they were responsible for—and the facilitation process should be more strategic and structured at the unit level. A successful factor in the implementation of the extensive mentoring programme seems to be a more step‐by‐step approach at each unit, adapted based on available resources such as the supervisors' knowledge, motivation and earlier experiences of similar roles. The nurse managers also requested an allocated budget to be able to implement this large project, which was announced by the hospital board. The hospital management needs to recognize activities serving to decrease nursing turnover (Selberg & Mulinari, 2021), and the mentoring programme could be seen as such an activity (Jangland et al., 2021). The programme is also in line with a statement by the International Learning Collaborative underscoring that hospitals need to address the pressing needs of registered nurses and have systems in place that care for their registered nurses and ensure professional development (Kitson et al., 2022). A financial investment, as described by the nurse managers, seems to be a reasonable request—and an important success factor—in the case of a large implementation project aiming to support registered nurses in their daily practice to prevent the current negative spiral of premature departure from the profession.

As pointed out earlier, facilitation is the driving force for successfully embedding, sustaining and disseminating an intervention, and a basic principle that needs to be in place to enable and support others to act (Harvey & Kitson, 2015). The innovation, that is the different supervision roles, was based on strong evidence (Ahlstedt et al., 2019; Brook et al., 2019; Hayes et al., 2012; Rudman et al., 2020), something the nurse managers and supervisors believed in and expected to be a positive innovation to solve the everyday nursing shortage in the hospital (Jangland et al., 2021). The project management team also had a communication strategy in place and arranged supervision sessions and follow‐up days for supervisors. However, the ‘real world’ at the units (e.g. lack of time to prioritize meetings, shortage of registered nurses on many shifts) led to a lack of support in a challenging assignment and hindered the further implementation.

The project management team seemed to have underestimated the supervisors' and nurse managers' need of support in the implementation process. Using the i‐PARIHS framework in the analysis revealed that it was crucial to have experienced facilitators in place throughout the organization. As pointed out by Harvey and Kitson (2016), given the scope and complexity of the facilitator role, it is crucial that the person who takes on that role can use facilitation methods and processes to enable and optimize implementation. A review shows that the PARIHS framework often tends to be applied retrospectively (Bergström et al., 2020), which was the case also in our study. A pilot study, using the i‐PARIHS framework as a guide ahead of the implementation (Kitson & Harvey, 2016), should ideally have been done as a first phase of the implementation, to test and evaluate this in a small‐scale project.

### 6.1 Strengths and limitations

One limitation is that we used a secondary analysis of data collected for another purpose (Jangland et al., 2021). However, the nurse managers and supervisors were invited to talk about the implementation processes during the interviews. The existing data were considered informative and could generate knowledge without burdening new participants (Beck, 2016). One strength is that we used the i‐PARIHS framework in our evaluation—a well‐known framework previously used in a range of settings (Bergström et al., 2020; Harvey & Kitson, 2016; Hunter et al., 2020) and which offers a facilitation checklist (Harvey & Kitson, 2015) (pp. 66–69). Using the framework in the evaluation aided understanding and clarification of the outcome of the implementation. The how‐to guide (i.e. the checklist) with many details to consider in the evaluation of the implementation supported the analysis and revealed successful factors as well as weaknesses in the implementation. However, we also identified a challenge in using the figures and checklist (Harvey & Kitson, 2015) in an integrated way, since they were inconsistent in presentation. Similar experiences were reported by Hunter et al. (2020). We discussed how to use the reflective questions presented in the checklist and added our interpretation and own examples. To ensure consistency in the analysis, the research team met on a regular basis for discussions. Another strength of the study is that participants from different units across the hospital were included, increasing transferability to different settings.

### 6.2 Clinical implications

The results could be used for developing the capacity for the implementation of the mentoring programme in hospital settings, as well as for increasing its sustainability. Clinical implications from the present study (presented in Table 4) could be transferred to similar implementation projects. Retention of registered nurses is a critical issue for healthcare organizations across the world and the mentoring programme—implemented in an organization with an infrastructure that enabled facilitators to keep units on track—is an activity that can prevent registered nurses' premature departure from the profession.

**TABLE 4:** Focus and activities in successful implementation of the mentoring programme.

Hospital management	Nurse manager	Supervisor
Develop a strategic plan for implementation, including continuous follow‐up. Offer support and guidance to nurse managers and experienced and novice facilitators across the hospital. Be available during the implementation process and later. Establish a management group including members from all levels in the organization (i.e. nursing leaders in the hospital management, nurse managers, supervisors and newly graduated nurses).	Take on the leadership role, including being aware of the role as facilitator or allocating a facilitator to the supervisors. Recruit competent, independent, committed and motivated senior nurses as supervisors. Support the implementation of the mentoring programme in accordance with the project description, adapted to each unit's particular needs, including a plan for evaluation.	Implement the mentoring programme in accordance with the project description, adapted to each unit's particular needs, including a plan for evaluation. Take full responsibility for the project at the unit level, work independently in collaboration with supervisor colleagues, report and reconcile in accordance with agreements or the needs of nurse managers. Participate in mentoring programme activities arranged by the management group at the hospital level (e.g. supervision on supervision, inspiration meetings and competence development courses for supervisors).

## 7 CONCLUSION

The implementation of the mentoring programme was a challenge for the organization. The organization needs to continue investing in implementation and adding a more structured facilitation process. A structured and prioritized management system should be established by the hospital board, including supportive leadership by nurse managers at the unit level. The supervisors have to take full responsibility for their part of the mentoring programme and have the skill to work independently. In addition, there is a need for experienced facilitators throughout the organization. This is crucial to achieve sustainability in the mentoring programme and ensure that the large investments of staff resources and money do not fizzle out.

## FUNDING INFORMATION

This research was funded by the Swedish Government through a grant supporting the region's transition‐to‐practice initiatives for newly graduated nurses. Some of the work was performed as part of the employment of the authors, Uppsala University.

## CONFLICT OF INTEREST STATEMENT

The authors have no conflict of interest.

## PEER REVIEW

The peer review history for this article is available at https://www.webofscience.com/api/gateway/wos/peer‐review/10.1111/jan.16041.

## Supporting information


Data S1:

## Data Availability

Access to data is restricted. People familiar with the context in which the study is carried out would be able to recognize the interviewees if they read the entire interviews. Therefore, with the support of GDPR and the Swedish Ethical Review Authority, we cannot make our data available.
